# Treatment of systemic sclerosis–associated pulmonary hypertension with and without interstitial lung disease and its impact on survival: a large cohort study of the German network for systemic sclerosis

**DOI:** 10.1093/rheumatology/keag155

**Published:** 2026-04-15

**Authors:** Marc Schmalzing, Pia Moinzadeh, Jithmi Weliwitage, Joerg Henes, Norbert Blank, Gernot Keysser, Gabriela Riemekasten, Ulf Mueller-Ladner, Margitta Worm, Aaron Juche, Alexander Kreuter, Jan Ehrchen, Laura Susok, Claudia Guenther, Gabriele Zeidler, Ilona Jandova, Tim Schmeiser, Christiane Pfeiffer, Ina Koetter, Annette Alberding, Joerg H W Distler, Nicolas Hunzelmann

**Affiliations:** Rheumatology/Clinical Immunology, Department of Internal Medicine II, University Hospital Würzburg, Würzburg, Germany; Dermatology and Venereology, University Hospital Cologne, Cologne, Germany; Institute for Medical Statistics and Bioinformatics, University of Cologne, Cologne, Germany; Interdisciplinary Rheumatology, Clinical Immunology and Autoimmune Diseases (INDIRA), University Clinic Tübingen, Tübingen, Germany; Inner Medicine Hematology, Oncology and Rheumatology, University Hospital Heidelberg, Heidelberg, Germany; Inner Medicine—Nephrology, Rheumatology, University Hospital Halle, Halle, Germany; Rheumatology, University Hospital Schleswig-Holstein, Luebeck, Germany; Rheumatology and Clinic Immunology, Kerckhoff Clinic, Bad Nauheim, Germany; Dermatology, Venereology and Allergology, University Hospital Charite Berlin, Berlin, Germany; Rheumatology and Clinic Immunology, Immanuel Hospital Berlin Buch, Berlin, Germany; Dermatology, Venereology and Allergology, Helios St. Elisabeth, Oberhausen, Germany; Dermatology, University Hospital Münster, Münster, Germany; Dermatology, Hospital Dortmund, Dortmund, Germany; Dermatology, University Hospital Carl Gustav Carus Dresden, Dresden, Germany; Department of Dermatology, University Hospital Tübingen, Interdisciplinary Rheumatology, Clinical Immunology and Autoimmune Diseases (INDIRA), Tübingen, Germany; Klinik für internistische Rheumatologie, spezielle Schmerztherapie und Osteologie des Johanniterkrankenhauses Treuenbrietzen, Treuenbrietzen, Germany; Rheumatology and Clinical Immunology, University Hospital Freiburg, Freiburg, Germany; Rheumatology, Rheumatologie im Veedel—Practice Dr. Schmeiser, Cologne, Germany; Clinic for Dermatology and Allergology, University Hospital Munich LMU, Munich, Germany; Rheumatology and Immunology, Hospital Bad Bramstedt, Bad Bramstedt, Germany; Department of Internal Medicine II–Internal Rheumatolgy, St. Josef Hospital Wuppertal, Wuppertal, Germany; Department of Dermatology, University Hospital Tübingen, Interdisciplinary Rheumatology, Clinical Immunology and Autoimmune Diseases (INDIRA), Tübingen, Germany; Rheumatology, University Hospital Düsseldorf, Duesseldorf, Germany; Dermatology and Venereology, University Hospital Cologne, Cologne, Germany

**Keywords:** pulmonary hypertension, systemic sclerosis, interstitial lung disease, survival, treatment

## Abstract

**Objectives:**

This study aimed to analyse survival in SS-associated pulmonary hypertension (SSc-PH) patients with or without interstitial lung disease (ILD) stratified by PH monotherapy and PH combination therapy, respectively.

**Methods:**

Among the 6003 patients registered with the German Network for Systemic Sclerosis (DNSS) registry, SSc patients with incident PH diagnosed after registry entry were included. Kaplan-Meier analysis was performed to estimate the overall survival, and the log-rank test was used to assess differences between groups stratified by the presence of ILD or PH treatment.

**Results:**

Of, the 384 patients who fulfilled the inclusion criteria, 271 had concomitant ILD (70.6%). The mean follow-up period was 8.69 ± 4.94 years. Forced vital capacity (FVC) and diffusing capacity of the lungs for carbon monoxide (DLCO) numerically decreased in PH patients with and without ILD and were significantly lower in patients with ILD (mean value of minimal FVC during follow-up: 70.79 ± 24.36%). There was no significant difference in the distribution of PH therapy categories (mono, dual, or triple therapy) between PH patients with and without ILD involvement (*P = *0.865). No survival difference was observed between the monotherapy and combination therapy groups (*P = *0.197). Subgroup analysis indicated that varying the treatment strategies did not significantly impact the survival outcomes of PH patients, whether or not ILD was present.

**Conclusion:**

SSc patients with ILD and incident PH are often treated with PH combination therapy in Germany. This treatment strategy seems feasible and does not worsen survival in PH patients, provided that ILD is not severe.

Rheumatology key messages

- In systemic sclerosis (SSc) patients, pulmonary hypertension (PH) is a known risk factor for morbidity and mortality.
- In the DNSS registry, the proportion of SSc patients with interstitial lung disease (ILD) and a new diagnosis of PH ever receiving combination PH treatment was high, although the international guidelines recommend cautious monotherapy.
- The patients did not have inferior survival compared with SSc-PH patients with ILD on continuous monotherapy.

## Introduction

Among the inflammatory rheumatic diseases, SSc is the one with the worst prognosis quoad vitam [1, 2]. Its pathogenesis is characterized by autoimmune inflammation, fibrosis, and endothelial damage leading to obliterative vasculopathy. The latter can present as RP, digital ulcerations or pulmonary arterial hypertension (PAH) [3]. Pulmonary hypertension (PH) is one of the leading causes of death in SSc, and survival prognosis seems to be particularly dismal if PH occurs together with interstitial lung disease (ILD) [4–6]. The prognosis for SSc-PH has improved during recent decades, in which there have been milestone developments in PAH medication and a more consistent implementation of screening strategies [7–14]. Whereas numerous studies could show an improvement in pulmovascular parameters and in the functional class of dyspnea, it has not so far been elucidated whether PAH medication has an influence on SSc-PH survival [8, 15].

To add to the complexity of the topic, several forms of PH have to be taken into consideration in SSc: PAH (group 1), PH associated with left heart disease (group 2), pulmonary hypertension associated with lung diseases (group 3), and chronic thrombo-embolic pulmonary hypertension (group 4) [16].

PH treatment algorithms differ substantially depending on the group of PH diagnosed in a patient, and diagnosis and treatment turns out to be particularly difficult if there is a mixture of PH groups in the individual patient. PH treatment in a patient with PH and ILD can lead to deterioration, because vasoactive drugs, which have been approved exclusively for PAH and not for PH in lung disease, can lead to hypoxemia by increasing the perfusion of poorly ventilated lung areas. The distinction in the individual PH patient with ILD, if PH is caused by PAH (group 1) with ILD as a comorbidity or by group 3 PH (PH primarily associated with the lung disease ILD), is often difficult to make. The European Society of Cardiology (ESC) and the European Respiratory Society (ERS) recommend up-front combination treatment [e.g. endothelin receptor antagonists (ERAs) plus phosphodiesterase-5-inhibitors (PDE5is)] in patients with PAH without cardiopulmonary comorbidities, whereas they recommend careful monotherapy in patients who have these comorbidities, e.g. with a PDE5i. They even advise against ERA in PH patients with idiopathic pulmonary fibrosis, and they advise against using PAH medication in patients with lung disease and non-severe PH [5]. As evidence for these recommendations, numerous phase 2 and phase 3 studies are cited, in which ERA or sildenafil were used to actually treat ILD, all with negative results. In the few randomized controlled trials (RCTs) on PAH medication for group 3 PH, clinical worsening occurred under ambrisentan and riociguat, including excess mortality [17–23]. Only one RCT on PAH medication in group 3 PH showed improvement for its primary end point (6 min walk distance) [24]. In this trial (INCREASE), patients received inhaled Treprostinil. A possible explanation would be the mode of application, by which vasodilation may mainly take place in well-ventilated lung areas [25].

Although the importance of PH and ILD, and particularly their combination, for patients with SSc, and their impact on prognosis are well known, only a few studies exist on drug treatment for PH in this population. There is a need to understand how these patients are treated in a real-world setting, whether treatment is done according to international guidelines, and whether these real-world treatment strategies are beneficial for patients as to their survival.

Therefore, the objective of this German national database study was to analyse the drug treatment of PH in patients with SSc, depending on the presence of ILD, to characterize these patients regarding standard parameters of disease severity for ILD and PH, respectively, and to determine whether patients within these disease subsets have inferior survival if they are not treated according to the international guidelines, with a focus on monotherapy vs combination therapy.

## Methods

### Study design

The DNSS registry is a patient registry of >6000 SSc patients from 25 clinical centres. The registry was started in 2003. In our study, a subset of DNSS patients’ records from 2003 to 16 January 2023 were included.

We considered the PH-directed treatments, endothelin receptor antagonists (ERA), phosphodiesterase-5-inhibitors (PDE-5i), soluble guanylate cyclase (sGC) stimulator and selexipag for the categorization of patients into therapy groups as monotherapy, dual therapy, or ‘triple-or-more’ combination therapies based on the number of treatments ever given simultaneously during the follow-up time. Due to the low patient number (*n* = 3), no separate category with quadruple combination was chosen. This classification allowed us to analyse the impact of different treatment strategies on patient outcomes. Prostanoids were not considered, since the case report form (CRF) did not distinguish between indication for prostanoid use, which in the majority of cases presumably was RP and digital ulcers, and not PH.

The definitions of diagnosis, date of SSc diagnosis, ILD, and PH are the same definitions used in prior studies based on the DNSS registry [2]. ILD was defined as SSc associated (SSc-ILD) when other possible causes of lung fibrosis were excluded and bilateral fibrosis was confirmed by chest radiograph or high-resolution CT (HRCT) scan. Since 2014, only a HRCT scan confirming ILD has been accepted. PH was defined as attributed by the treating physician in the CRF. As a prerequisite for this attribution, the CRF glossary demanded an increase of mean pulmonary arterial pressure of >25 mm at rest, evaluated by right heart catheterization.

### Inclusion and exclusion criteria

**Figure 1:**
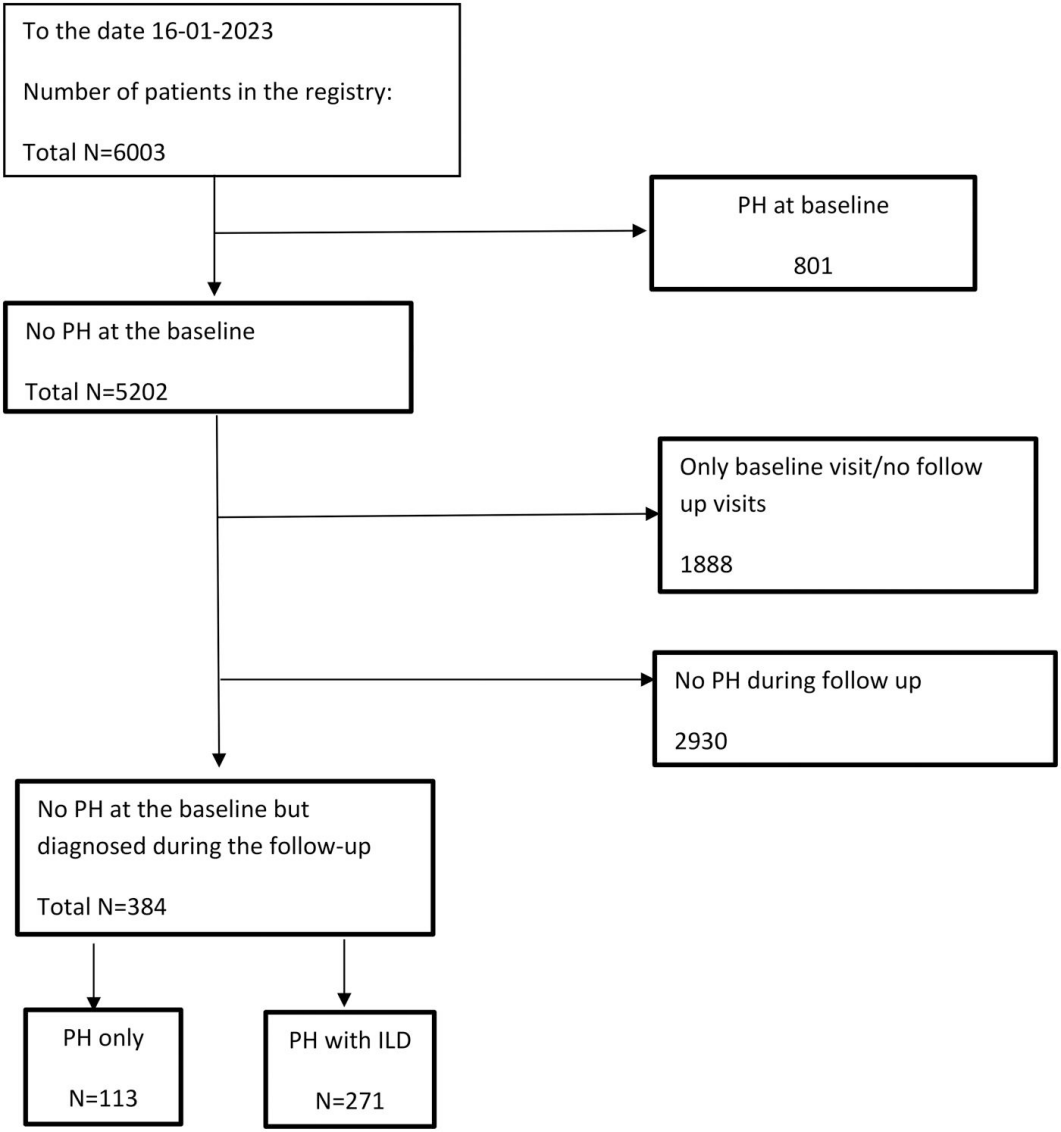
Flowchart: selection process of patients from the German Network for Systemic Sclerosis (DNSS) registry to include patients with a new diagnosis of pulmonary hypertension (PH) and at least one follow-up visit, and stratification by the presence or absence of interstitial lung disease (ILD)

Eligible patients included patients of the DNSS registry aged ≥18 years with a diagnosis of SSc according to the classification criteria published by the ACR and EULAR [2]. Classification of patients with dcSSc *vs* lcSSc was performed by the criteria established by LeRoy *et al*. [26]. Since the primary objective of this study was to analyse PAH treatment effects on survival after PH diagnosis, only patients who had no PH at baseline but developed PH during follow-up were included in our study analysis cohort. This selection allowed us to best ensure that all patients in our cohort were comparable in terms of PAH treatment patterns. The flowchart of selecting the study cohort is depicted in Fig. 1. The selected cohort was stratified into subgroups based on whether patients had ILD. Patients were attributed to the group ‘PH with ILD’, if ILD diagnosis was made at the same study visit as when PH diagnosis was made or before that. In the group ‘PH only’, no ILD was diagnosed at any of the recorded visits. According to clinical practice, ILD severity was categorized as follows: mild [forced vital capacity (FVC) ≥ 60%], moderate (FVC 40% to 60%), and severe (FVC < 40%) [27].

### Survival analysis

Kaplan–Meyer survival analysis was performed for the time from the first recording of PH (baseline) to the date of death (any cause) or the end of follow-up (limited to the last visit) and compared in the patient subgroups of ILD involvement and therapy category. Survival analyses included only the actual events (death), while censoring patients at their last known follow-up.

### Statistical analysis

Demographic and baseline characteristics are presented as applicable by mean, s.d., or count and percentage. For the comparison of scale parameters, we used the independent sample *t* test for normally distributed data and the Mann–Whitney *U* test for non-normally distributed data, based on the assessment of normality. The Pearson χ^2^ test was used to compare categorical parameters.

Kaplan–Meier analysis was performed to estimate the overall survival. Survival curves were generated and compared using the log-rank test to assess differences between groups.

All reported *P* values are two-sided, and *P* < 0.05 was considered statistically significant. Unless otherwise indicated, CIs are 95% CIs. Because the analyses were regarded as explorative, we did not adjust for multiple testing. Calculations and figures were undertaken using SPSS [28.0.1.1(14); IBM Corp] and R (version 4.4.0; R Foundation for Statistical Computing).

## Results

### Patient baseline characteristics

As of 16 January 2023, 6003 patients were included in the DNSS registry. Of these patients, 19.7% had PH at baseline or developed PH during follow-up—801 (13.3%) already had PH at the baseline visit, and that group were not included for further analysis. Of the remaining 5202 patients, 1888 had only one documented visit and no annual follow-up visits. Of the remaining 3314 patients, 384 (11.6%) developed PH during follow-up. Of these PH patients, 271 had concomitant ILD (70.6%).

Only the 384 PH patients who developed PH during follow-up were included in further analyses. Table 1 contains the baseline characteristics of the patients.

**Table 1:** Demographics and baseline characteristics of all included patients with pulmonary hypertension (PH), PH patients without interstitial lung disease (ILD) (=PH only group) and PH patients with ILD (=PH with ILD group).

	All patients	PH only group	PH with ILD group	*P*-value
	*N* (%) or mean ± s.d. (*n*)	*N* (%) or mean ± s.d. (*n*)	*N* (%) or mean ± s.d. (*n*)	
Number of patients	384	113	271	
Gender (male)	31/383 (18.5%)	16/113 (14.2%)	55/270 (20.4%)	0.154
Age at first entry (y)	57.46 ± 12.89 (383)	60.77 ± 11.84 (113)	56.07 ± 13.07 (270)	0.001
Age at SSc diagnosis (y)	50.69 ± 14.15 (383)	54.58 ± 13.65 (113)	49.06 ± 14.06 (270)	<0.001
SSc disease type				<0.001
Limited	193 (51.2%)	79 (71.2%)	114 (42.8%)	
Diffuse	141 (37.4%)	11 (9.9%)	130 (48.9%)	
Overlap	43 (11.4%)	21 (18.9%)	22 (8.3%)	
Disease duration (y)	15.47 ± 9.39 (384)	14.13 ± 9.24 (113)	16.02 ± 9.41 (271)	0.071
Baseline ILD involvement (yes)	156/384 (40.6%)	0/113 (0%)	156/271 (57.6%)	<0.001
mRSS	9.93 ± 8.96 (355)	6.64 ± 6.23 (104)	11.29 ± 9.56 (251)	<0.001
mRSS > 10	138/355 (38.9%)	22/104 (21.2%)	116/251 (46.2%)	<0.001
mRSS > 15	79/355 (22.3%)	10/104 (9.6%)	69/251 (27.5%)	<0.001
mRSS > 20	45/355 (12.7%)	5/104 (4.8%)	40/251 (15.9%)	0.004
ESR (mm/h)	21.49 ± 17.77(325)	16.80 ± 13.08 (89)	23.26 ± 18.97 (236)	0.003
Scl-70	132/381 (34.6%)	13/112 (11.6%)	119/270 (44.1%)	<0.001
Centromere	141/381 (37.0%%)	70/112 (62.5%)	71/269 (26.4%)	<0.001
Other antibodies	131/383 (34.2%)	34/113 (30.1%)	97/270 (35.9%)	0.272
Glucocorticoids	138/371 (37.2%)	29/108 (26.9%9	109/263 (41.4%)	0.008
Immunosuppressive medication	140/371 (37.7%)	31/108 (28.7%)	109/263 (41.4%)	0.021
Smoking				0.270
No smoker	134/218 (61.5%)	37/67 (55.2%)	97/151 (64.2%)	
Actual smoker	15/218 (6.9%)	7/67 (10.5%)	8/151 (5.3%)	
Ex-smoker	69/218 (31.7%)	23/67 (34.3)	46/151 (30.5%)	
NYHA functional class				<0.001
NYHA II	100/217 (46.1%)	43/64 (67.2%)	57/153 (37.3%)	
NYHA III	91/217 (41.9%)	18/64 (28.1%)	73/153 (47.7%)	
NYHA IV	26/217 (12.0%)	3/64 (4.7%)	23/153 (15.0%)	
Oxygen-dependent	43/253 (17.0%)	4/76 (5.3%)	39/177 (22.0%)	0.001
Death during follow-up	51/384 (13.3%)	9/113 (8.0%)	42/271 (15.5%)	0.047
Follow-up time (y)	8.69 ± 4.94 (384)	7.91 ± 4.86 (113)	9.02 ± 4.95 (271)	0.044

All variables were compared between the PH only group and the PH with ILD group and tested for statistical significance. mRSS: modified Rodnan Skin Score; NYHA: New York Heart Association.

PH-patients without ILD (PH only) were significantly older, had a higher prevalence of a limited cutaneous subtype, had a lower mean mRSS and lower mean ESR, had a higher prevalence of ACA instead of anti-SCl70 antibodies, had a lower functional New York Heart Association (NYHA) class, and less oxygen dependency compared with patients with ILD (PH with ILD). These differences were found to be statistically significant. The death rate was significantly higher in patients with ILD compared with those without ILD (15.5% *vs* 8.0%, *P = *0.047). The percentage of patients under immunosuppressive therapy in the PH with ILD group was significantly higher than in patients who had only PH without ILD (PH with ILD *vs* PH only, 41.4% *vs* 28.7%, *P = *0.025). No significant differences were observed as to gender, disease duration or smoking status (Table 1).

There were no patients with severe ILD in our cohort (defined by FVC < 40% predicted), and most patients had mild ILD (73.4%), while 26.6% had moderate ILD.

### Cardiopulmonary disease parameters during follow-up

When comparing the disease parameters of ILD and PH during follow-up, different lengths of follow-up time and different numbers of follow-up visits in this real-world study had to be taken into account. Therefore, for each parameter, the mean value at baseline, the average value of all follow-up visits, and the clinically worst value were compared (Table 2). Regarding lung function tests, the reduction in FVC and diffusing capacity of the lungs for carbon monoxide (DLCO) was comparable between PH-patients with and without ILD. Average and worst values during follow-up were significantly lower in patients with ILD, as expected.

**Table 2:** Disease parameters of all included patients with pulmonary hypertension (PH), PH patients without interstitial lung disease (ILD) (=PH only group) and PH patients with ILD (=PH with ILD group).

	All patients	PH only group	PH with ILD group	*P*-value
	*N* (%), mean ± s.d. (*n*) or median [IQR]	*N* (%), mean ± s.d. (*n*) or median [IQR]	*N* (%), mean ± s.d. (*n*) or median [IQR]	
Number of patients	384	113	271	
Follow-up time	8.69 ± 4.94 (384)	7.91 ± 4.86 (113)	9.02 ± 4.95 (271)	0.045
Disease parameters of interstitial lung disease				
FVC (%pred.) baseline	86.11 ± 24.74 (228)	95.34 ± 22.44 (68)	82.18 ± 24.70 (160)	<0.001
FVC (%pred.) average	83.21 ± 22.87 (228)	93.56 ± 21.02 (68)	78.81 ± 22.26 (160)	<0.001
FVC (%pred.) min	75.68 ± 24.83 (228)	87.19 ± 22.13 (68)	70.79 ± 24.36 (160)	<0.001
DLCO (%pred) baseline	66.26 ± 21.30 (369)	74.70 ± 22.00 (109)	62.73 ± 20.01 (260)	<0.001
DLCO (%pred) average	59.47 ± 18.35 (369)	67.34 ± 18.01 (109)	56.16 ± 17.49 (260)	<0.001
DLCO (%pred) min	48.06 ± 19.38 (369)	56.87 ± 18.43 (109)	44.36 ± 18.59 (260)	<0.001
Disease parameters of pulmonary hypertension				
NTproBNP (ng/l, baseline)	5.46 [4.25, 6.42]	5.58 [3.96, 6.52]	5.01 [4.40, 6.18]	0.634
NTproBNP (ng/l, max)	5.93 [4.44, 7.23]	6.40 [5.34, 7.06]	5.88 [5.03, 8.74]	0.342
NTproBNP (ng/l, average)	5.73 [4.20, 7.95]	5.84 [5.10, 6.68]	5.60 [4.73, 6.87]	0.517
PAP sys (mmHg) baseline	42.26 ± 17.28 (199)	39.62 ± 14.98 (61)	43.43 ± 18.13 (138)	0.125
PAP sys (mmHg) average	37.63 ± 14.58 (199)	35.69 ± 12.61 (61)	38.48 ± 15.33 (138)	0.183
PAP sys (mmHg) max	42.26 ± 17.28 (199)	39.62 ± 14.98 (61)	43.43 ± 18.13 (138)	0.125
LVEF (%pred.) baseline	56.88 ± 9.26 (184)	58.98 ± 9.35 (54)	56.01 ± 9.11 (130)	0.051
LVEF (%pred.) average	59.62 ± 7.23 (184)	60.95 ± 88.87 (54)	59.07 ± 6.38 (130)	0.162
LVEF (%pred.) min	56.88 ± 9.26 (184)	58.98 ± 9.35 (54)	56.01 ± 9.11 (130)	0.051
PAWP (mmHg) baseline	11.88 ± 3.87 (49)	12.20 ± 4.48 (15)	11.74 ± 3.63 (34)	0.727
PAWP (mmHg) average	11.38 ± 3.29 (49)	11.43 ± 3.66 (15)	11.36 ± 3.18 (34)	0.945
PAWP (mmHg) max	11.98 ± 3.88 (49)	12.20 ± 4.48 (15)	11.88 ± 3.66 (34)	0.811
Mean PAP (mmHg) baseline	29.36 ± 12.26 (72)	28.85 ± 13.77 (20)	29.58 ± 11.77 (52)	0.836
Mean PAP (mmHg) average	28.61 ± 12.02 (72)	28.29 ± 13.20 (20)	28.73 ± 11.66 (52)	0.449
Mean PAP (mmHg) max	29.37 ± 12.26 (72)	28.85 ± 13.77 (20)	29.58 ± 11.77 (52)	0.418

For all follow-up parameters, the baseline value, the average value of all follow-up visits, and the worst value of all follow-up visits are presented and tested for statistical significance, comparing the PH only group *vs* the PH with ILD group. DLCO: diffusing capacity for **c**arbon mon**o**xide; FVC: forced vital capacity; NTproBNP: N-terminal Pro-Brain Natriuretic Peptide; peroxidase–antiperoxidase (sys): (systolic) pulmonary arterial pressure; LVEF: left ventricular ejection fraction; PAWP: pulmonary arterial wedge pressure; PAP: pulmonary arterial pressure.

On the contrary, parameters of PH at baseline or follow-up did not differ significantly between patients with or without ILD, with the exception of average NTproBNP, which was significantly higher in PH patients with ILD (1347.66 ± 3417.62 *vs* 714.03 ± 967.50; *P = *0.041). In both subgroups NTproBNP increased, and PAPsys on echocardiogram decreased on average, whereas mean peroxidase–antiperoxidase on right heart catheter and left ventricular function on echocardiogram remained stable. As expected, right heart catheter–being the more invasive procedure—was performed and documented less frequently compared with echocardiogram as the follow-up diagnostic procedure for PH status.

Importantly, although patients with ILD had significantly worse lung function parameters (see Table 2), FVC as the most important parameter for restrictive pulmonary disease was still relatively high and corresponded to a merely mild restrictive lung disease.

### PH-directed therapy

The numbers of patients treated with various PH-directed drugs are given in Table 3. The percentages of patients treated with ERA and PDE5i were significantly higher in PH patients with ILD (ERA: 51.4% *vs* 30.9%; PDE5i: 43.3% *vs* 29.9%). Only 5.9% of patients from the total cohort were treated with soluble guanylat cyclase (sGC)-stimulator, and significant change due to ILD involvement was not seen. Because of incomplete records (due to Selexipag having a high rate of missing data), only patients who confirmedly received the medication were included in the evaluable population.

**Table 3:** Pulmonary arterial hypertension (PAH) therapies of all included patients with pulmonary hypertension (PH), PH patients with no interstitial lung disease (ILD) (=PH only) and PH patients with ILD (= PH with ILD group).

	All patients	PH only group	PH with ILD group	*P*-value
	*N* of evaluable patients (%)	*N* of evaluable patients (%)	*N* of evaluable patients (%)	
Number of patients	384	113	271	
ERA	164/363 (45.2%)	34/110 (30.9%)	130/253 (51.4%)	<0.001
PDE5i	139/354 (39.3%)	32/107 (29.9%)	107/247 (43.3%)	0.018
Selexipag	77/77 (100%)	25/25 (100%)	52/52 (100%)	–
sGC-stimulator	15/253 (5.9%)	4/76 (5.3%)	11/177 (6.2%)	1.000
Treatment category				0.865
Monotherapy	143/252 (56.7%)	37/62 (59.7%)	106/190 (55.8%)	
Dual therapies	78/252 (31.0%)	18/62 (29.0%)	60/190 (31.6%)	
Triple or more therapies	31/252 (12.3%)	7/62 (11.3%)	24/190 (12.6%)	

Patients were attributed to treatment categories if they ever received this therapy. Frequencies of treatment categories were compared between the PH only group and the PH with ILD group and tested for statistical significance. ERA: endothelin receptor antagonist; PDE5i: phosphodiesterase 5 inhibitor; sGC: soluble guanylate cyclase.

**Figure 2:**
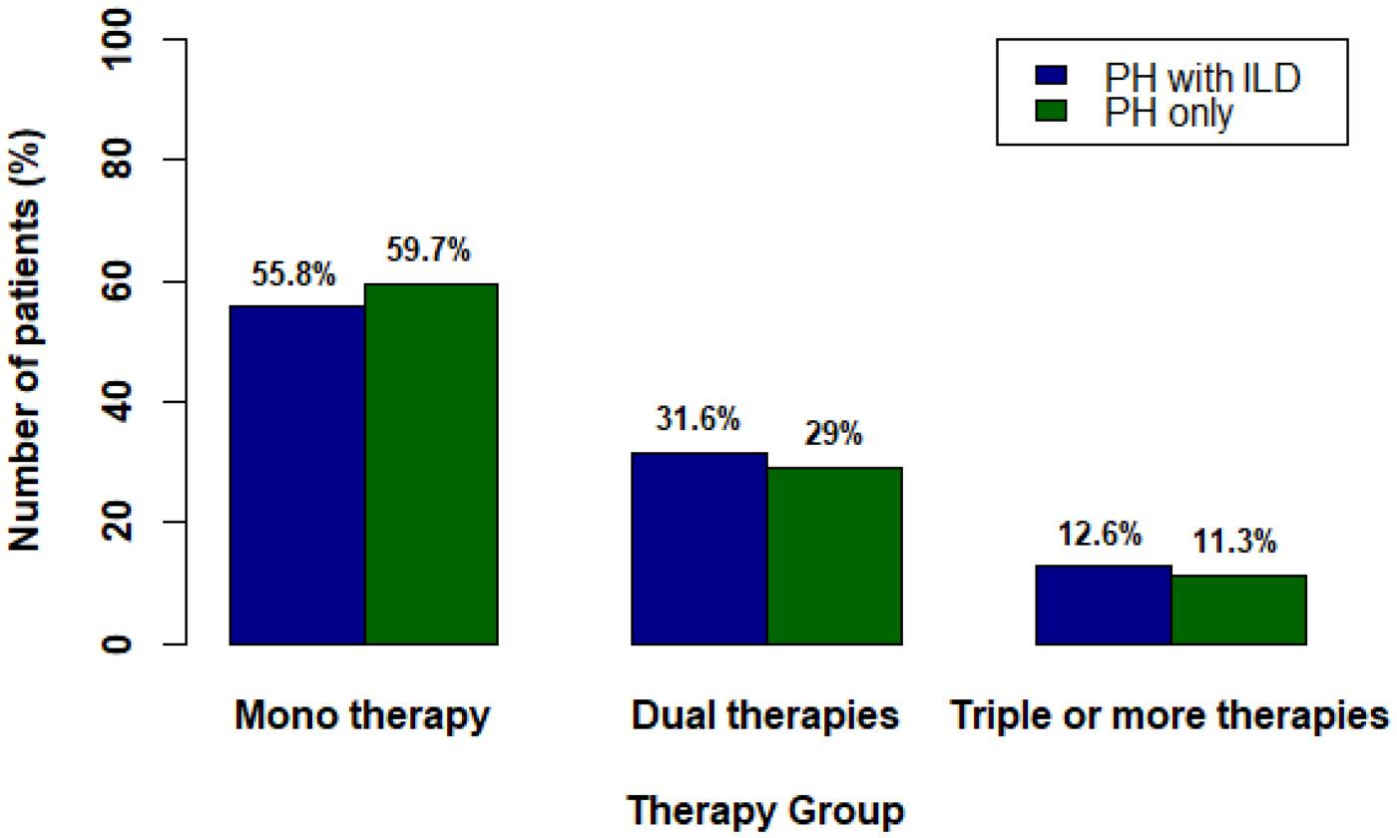
Distribution of pulmonary arterial hypertension-therapy groups (monotherapy, dual combination therapy, triple or more combination therapy) in pulmonary hypertension (PH) patients stratified by the presence or absence of interstitial lung disease (ILD)

Of note, there was no significant difference in the distribution of therapy categories (mono, dual, or triple therapy) between PH patients with and without ILD involvement (*P = *0.865). The distribution of treatment categories is depicted in Fig. 2.

### Survival analysis

**Figure 3:**
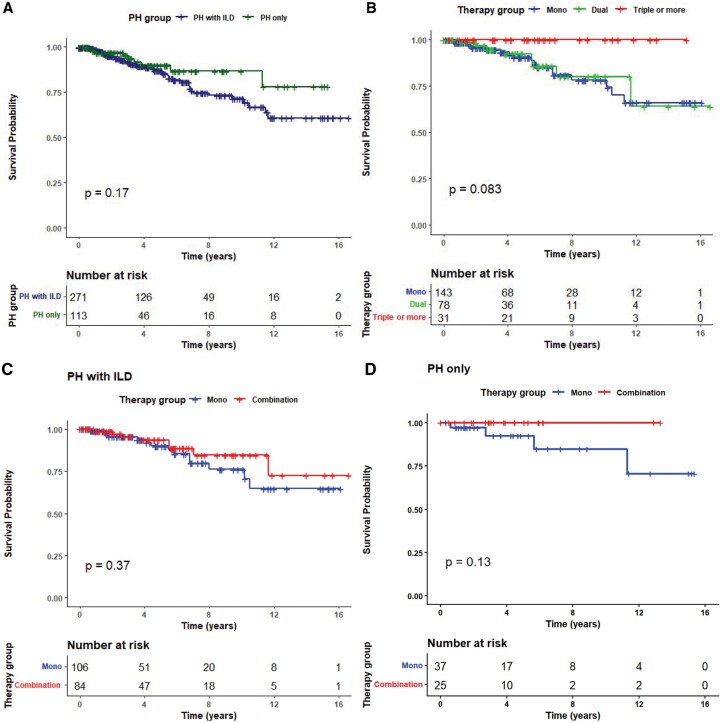
Kaplan–Meier survival curves. (A) Kaplan–Meier survival curves in patients with SSc, stratified by the presence of interstitial lung disease (ILD) with pulmonary hypertension (PH), or PH only without ILD. (B) Kaplan–Meier survival curves in patients with SSc, stratified by pulmonary hypertension (PH)-therapy group. (C) Kaplan–Meier survival curves in SSc patients with PH with ILD, stratified by PH-therapy group. (D) Kaplan–Meier survival curves in SSc patients with PH without ILD (PH only), stratified by PH-therapy group

Kaplan–Meier survival analysis was performed to compare the survival distributions of PH patients with ILD involvement *vs* those without (Fig. 3A–D). The analysis revealed no significant difference in survival between the two groups, as indicated by the log-rank test (*P = *0.169). The log-rank test of survival distributions across the therapy groups of PH patients regardless of ILD involvement showed that patients with triple or more therapies had better survival (no events, 100% survival) compared with both monotherapy (*P = *0.025) and dual therapy (*P = *0.034) groups. However, there was no significant difference between the monotherapy and dual therapy groups (*P = *0.851).

We performed a subgroup analysis of the survival distributions of the therapy groups, considering the ILD involvement. In the PH only patient group, only monotherapy patients experienced events with a 10.8% rate (4/37) during the study period, while other therapy groups had no events. However, there were no statistically significant differences between patient survival in monotherapy PH patient vs dual therapy PH patients (*P = *0.278) or between monotherapy PH patients and triple or more therapies PH patients (*P = *0.288).

Deaths occurred in the cohort of PH patients with ILD treated with monotherapy (13.2%, 14/106) and dual therapy (13.3%, 8/60), but in the group with triple or more therapies there were no deaths. The survival distribution of monotherapy PH patients with ILD showed a significant difference to the triple-or-more therapies group (*P = *0.046).

Since no events occurred in the triple-or-more therapies group during the follow-up period, and due to the clinical similarity with the dual therapy group according to disease parameters, these two groups were merged into a single ‘combination therapy’ group. No survival difference was observed between the monotherapy and combination therapy groups (*P = *0.197). Subgroup analysis indicated that varying PH treatment strategies did not significantly impact the survival outcomes of PH patients whether or not ILD was present (Table 4).

**Table 4:** Kaplan–Meier analysis and survival rates of pulmonary hypertension (PH) patients by interstitial lung disease (ILD) involvement and therapy categories.

Covariate		Total *N*	Events	Median	Survival rates with CI		Mean (years) [95% CI]	*P* value (log-rank)
					at 5 years	at 10 years		
PH group	PH only	113	9	NR	89.79% [82.69, 97.51]	86.59% [77.65, 96.56]	13.35 [12.12,14.59]	0.169
	PH with ILD	271	42	NR	86.99% [82.14, 92.13]	71.44% [63.24, 80.71]	12.66 [11.62, 13.71]	
Therapy group	Mono	143	18	NR	90.55% [84.68, 96.83]	78.16% [68.10, 89.72]	12.99 [11.73, 14.26]	0.851
	Dual Triple or more	78	8	NR	92.36% [85.33, 99.97]	80.05% [66.37, 96.54]	13.48 [11.41, 15.55]	
		31	0	NR	–	–	–	–
Therapy group	Mono	143	18	NR	90.55% [84.68, 96.83]	78.16% [68.10, 89.72]	12.99 [11.73, 14.26]	0.197
	Combination	109	8	NR	94.85% [90.00,99.96]	86.76% [77.13, 97.61]	14.59 [13.19, 15.99]	
PH only	Mono	37	4		92.35% [82.48, 100.0]	84.66% [68.99, 100.0]	–	–
	Combination	25	0		–	–	–	
PH with ILD	Mono	106	14	NR	89.98% [82.99, 97.57]	76.05% [64.04, 90.32]	12.83 [11.32, 14.33]	0.373
	Combination	84	8	NR	93.44% [87.37, 99.93]	84.49% [73.77, 96.79]	14.18 [12.53, 15.83]	

NR: not reached.

There was even a trend towards better mean survival in patients with PH and ILD under PH combination therapy compared with PH monotherapy (14.18 years *vs* 12.83 years, *P = *0.373; the 10-year survival rate being 84.49% *vs* 76.05%, Table 4), and this trend could still be found in both subgroups with mild ILD (14.56 years *vs* 13.50 years) and with moderate ILD (12.83 *vs* 11.53 years). Also, when using the FVC cut-off of 70% according to the Goh-algorithm [28] to distinguish between limited and extensive ILD, similar trends were found in both groups, without reaching statistical significance (data not shown). No influence of immunosuppressive or glucocorticoid treatment on survival was found statistically (data not shown).

## Discussion

In this study, the DNSS registry provides comprehensive real-world data on the prevalence of PH and ILD in SSc and the use of PAH medications in these patients in German-speaking countries. To our knowledge, this is the largest study describing the use of vasodilator therapy in SSc patients with incident PH with or without ILD (DNSS: *N* = 384, REHAP: *N* = 220 [29], Australia: *N* = 164 [15]. Furthermore, this is the first study investigating the impact of vasodilator monotherapy *vs* combination therapy on survival in SSc patients with PH and ILD.

A total of 384 SSc patients with new PH during follow-up were selected from over 6000 patients in the DNSS registry to achieve a homogeneous study population with incident PH for survival analysis stratified by PH treatment strategy. Of these, 271 had ILD. Significant differences in the baseline characteristics of the ILD patients compared with patients without ILD confirmed well-known risk factors for ILD in SSc: diffuse cutaneous subtype, high mRSS, and Scl70 positivity. The cardiopulmonary disease parameters at baseline only differed as to worse lung function tests in patients with ILD, as expected, whereas the parameters of PH were not significantly different. This would argue for a similar extent of pulmovascular disease in patients with or without ILD in our cohort. Interestingly, the parameters for restrictive lung disease were only mildly decreased in patients with ILD on average, and even the mean minimum value of FVC during follow-up was above 70% of predicted in this patient group. This raises the possibility that a substantial proportion of DNSS patients classified as PH-ILD may have had predominantly PAH (group 1) with coexisting mild ILD, rather than PH primarily driven by advanced parenchymal lung disease (group 3).

This may also explain the comparable frequencies of vasodilator monotherapy and combination therapy, respectively, in patients with ILD and patients without ILD. Of note, in our study >40% of patients with ILD ever received combination therapy, numerically even more than patients without ILD. Our study is not suited to elicit the reason why patients often remained on monotherapy contrary to recommendations in the guidelines. It has to be kept in mind that the study population was selected before the current guidelines were published.

In our study, there was a trend for longer survival in PH-patients without ILD compared with PH patients with ILD, although this difference was not statistically significant. Of note, this survival difference was significant in the larger cohort taken from the same registry (DNSS) published by Moinzadeh and colleagues [5]. The results from other studies are conflicting, but most show a particularly dismal prognosis for patients with PH who also have ILD [30].

In a recent Spanish study using the REHAP and RESCLE registry data including 220 SSc-PH patients with information on ILD, 92 (59.8%) of those with both ILD and PH received up-front combination therapy. Notably, the survival outcomes were similar between SSc-PH patients with and without ILD, suggesting that an intensive treatment strategy may mitigate the typically poorer prognosis associated with ILD [31].

In a single-centre analysis of the John Hopkins registry of 504 SSc-PH patients, SSc patients with group 1 PH had improved transplant-free survival compared with past decades (diagnosed between 1999 and 2010, and diagnosed between 2010 and 2021, respectively), whereas the prognosis for groups 2 or 3 PH did not improve [8]. The authors speculated that this difference could partly be explained by the fact that the more recent cohort of group 1 PH patients received vasodilator combination treatment more often than the cohort in the past. In addition, more screening in group 1 probably led to an earlier diagnosis and hence a more favourable outcome.

In the ASPIRE SSc registry, survival in PAH was superior to that of group 3 PH. However, patients with mild ILD showed a similar outcome to that of patients without ILD [29].

Morrisroe and colleagues describe a smaller Australian SSc-PH cohort (164 SSc patients with incident PH). Patients with PAH without ILD also had a high frequency of up-front monotherapy (59.8% *vs* 52.8% in PH-ILD), and more than a third of patients with PH-ILD with less extensive ILD had combination therapy (34.7% *vs* 32.6% in PAH without ILD) [15]. The study separately analysed the data for 29 patients with PH and extensive ILD. Of these, only 3.5% received initial combination therapy. Thus, physicians seem to be more inclined to treat PH patients with mild restrictive lung disease with combination therapy than to treat patients with extensive ILD with combination therapy. The Australian study did not show a survival advantage of vasodilator treatment for PH-patients with ILD, but did not compare survival stratified for monotherapy *vs* combination therapy.

In our study, this stratification was undertaken, and interestingly, there was a trend for longer survival in PH-ILD patients under combination therapy, without reaching statistical significance. At the very least, the fear that more intensive vasodilator therapy shortens survival by increasing hypoxemia in ILD patients was not confirmed, but rather the opposite result was found. A possible explanation may be the mild degree of ILD in our cohort, which all the same suffered from severe pulmovascular disease, and the fact that our ILD patients rather had ILD and PAH (group 1) than PH due to lung disease (group 3). Nevertheless, the trend towards better survival under PH combination treatment was found both in patients with mild ILD and in patients with moderate ILD. Furthermore, no influence of immunosuppressive medication was seen statistically. However, the subgroups were too small to analyse these factors in detail or in a multivariate manner. This should be subject of larger future studies.

The finding that there were no deaths in PH patients with three or more PH therapies in combination is astonishing at first sight, since one would assume that this is the group with most severe PH and hence worst prognosis. This can possibly be explained by a better health status and less comorbidities, which would make a combination therapy more feasible and therefore more effective.

Taken together, our study confirms the considerable percentage of vasodilator combination treatment used in PH-SSc patients with ILD in the aforementioned studies, and seems to show more clearly than before that this therapeutic strategy may even have a favourable effect on survival.

### Study limitations

From the data in the CRF, a distinction between PAH with mild ILD as a comorbidity (group 1) on the one hand and PH associated with lung disease (group 3) on the other hand, was not possible. The high prevalence of patients with ACA–known to be a favourable risk factor for ILD [6, 32]—and the high mean FVC in our PH with ILD cohort, would speak for the former. Thus, it has to be pointed out that our study results on the survival of PH patients with ILD almost exclusively refer to patients with PAH (group 1) and very mild ILD and not to patients with group 3 PH.

The old version of our CRF, which was mainly used during the study period, does not allow distinction of the indication for prostanoids. Presumably, most patients received prostanoids because of RP or digital ulcers. Therefore, prostanoids as PH treatment could not be analysed.

Furthermore, the CRF did not capture all of the different groups of PH, so PH associated with left heart failure or chronic thromboembolic pulmonary hypertension (CTEPH) could not be recorded. However, PH in left heart failure probably did not play a major role in our cohort, since the mean pulmonary wedge pressure was low.

Not all standard parameters of PH severity were recorded, such as pulmonary resistance or cardiac output. The current version of the DNSS CRF was introduced in 2023 and contains these missing values and stratifications. Thus, future analyses of the DNSS registry may well be able to fill the data gaps mentioned.

In conclusion, this is the largest study in SSc-PH patients comparing survival of patients with or without ILD, depending on whether they ever received vasodilator monotherapy or combination therapy. The trend for better survival under combination therapy even in PH-SSc patients with ILD, which was mild in most cases in our cohort, would indicate the necessity to thoroughly analyse the aetiology of PH in SSc patients, especially in patients with a mixed aetiology of PH, and to treat consistently according to this analysis.

The DNSS registry is a patient registry of >6000 SSc patients from 25 clinical centres. The registry was started in 2003 and was approved by the Ethics Committee of the coordinating centre of University Hospital Cologne (approval No. 04–037). Informed written consent was obtained from all patients in the study.

## Data Availability

The data from the German Registry of Systemic Sclerosis (DNSS) underlying this article are stored digitally at the University of Cologne, Germany, and will be shared on reasonable request to the corresponding author. For all data-related queries, please contact the lead author (M.S.).
